# Flight capacities of yellow-legged hornet (*Vespa velutina nigrithorax*, Hymenoptera: Vespidae) workers from an invasive population in Europe

**DOI:** 10.1371/journal.pone.0198597

**Published:** 2018-06-08

**Authors:** Daniel Sauvard, Vanessa Imbault, Éric Darrouzet

**Affiliations:** 1 Institut national de la recherche agronomique, UR 633, Zoologie forestière, 2163 avenue de la pomme de pin, CS 40001 Ardon, 45075 Orléans cedex 2, France; 2 Institut de recherche sur la biologie de l’insecte, UMR CNRS 7261, Université François Rabelais, Faculté des Sciences, Parc de Grandmont, 37200 Tours, France; Philipps-Universitat Marburg Fachbereich Biologie, GERMANY

## Abstract

The invasive yellow-legged hornet, *Vespa velutina nigrithorax* Lepeletier, 1836 (Hymenoptera: Vespidae), is native to Southeast Asia. It was first detected in France (in the southwest) in 2005. It has since expanded throughout Europe and has caused significant harm to honeybee populations. We must better characterize the hornet’s flight capacity to understand the species’ success and develop improved control strategies. Here, we carried out a study in which we quantified the flight capacities of *V. velutina* workers using computerized flight mills. We observed that workers were able to spend around 40% of the daily 7-hour flight tests flying. On average, they flew 10km to 30km during each flight test, although there was a large amount of variation. Workers sampled in early summer had lower flight capacities than workers sampled later in the season. Flight capacity decreased as workers aged. However, in the field, workers probably often die before this decrease becomes significant. During each flight test, workers performed several continuous flight phases of variable length that were separated by rest phases. Based on the length of those continuous flight phases and certain key assumptions, we estimated that *V. velutina* colony foraging radius is at least 700 m (half that in early summer); however, some workers are able to forage much farther. While these laboratory findings remain to be confirmed by field studies, our results can nonetheless help inform *V. velutina* biology and control efforts.

## Introduction

Biological invasions have been on the rise for several decades, and this increase is now accelerating [1]. They are a major threat to biodiversity [2], and certain invaders are among the world’s most dangerous pests [3–5]. We need to better understand the biology of invasive species, especially that displayed in the non-native range(s), to predict their establishment, anticipate their spread, and limit their impacts. A large number of invasive species are insects [1]. Most can fly, and this trait plays an important role in their establishment. It affects dispersal, diet, survival, and reproduction, which means that it also affects the degree of damage an insect invader can cause.

The yellow-legged hornet, *Vespa velutina nigrithorax* Lepeletier, 1836 (Hymenoptera: Vespidae), was first detected in southwestern France in 2005, but it had likely been introduced in 2004, if not earlier [6, 7]. It is native to the continent of Asia, where its range extends from northern India to China and includes Southeast Asia. It seems that a few females or even just a single one were introduced into France from eastern China [8]. Having arrived in Europe, the species progressively spread in all directions. In 2016, it was present throughout France and had also been observed in Spain, Portugal, Italy, Germany, Belgium, and the United Kingdom [9]. This invasive species is causing major damage to the already threatened bee industry [10–13]. Moreover, *V. velutina* has likely had an appreciable, albeit poorly characterized, impact on insect biodiversity. This impact may be direct, a result of the hornet’s predatory lifestyle, or indirect, a consequence of hornet control efforts (current trapping methods lack specificity [14]).

At present, there is no satisfactory approach for dealing with *V. velutina*. It does not cause major economic problems in its native range, probably because indigenous honeybees ewtextcan deal with hornet predation [15–17]. Consequently, *V. velutina* has not been extensively studied in Asia. Even if its recent invasion in Europe has prompted research on the hornet’s biology [13], several knowledge gaps remain. For example, the species’ flight capacity is a key component of its invasive impact and will affect potential control strategies. In *V. velutina*, as in other social Vespidae, flight is crucial. It is the basic mode of locomotion outside the nest and frames the foraging behavior (workers, i.e. non-breeding females), reproductive behavior (males and breeding females), and dispersal (breeding females). Workers flight capacity influences in particular colony foraging radius, which is a critical determinant of resource acquisition (depending on local environmental conditions) and thus colony success. At present, one of the most common control techniques is identifying and destroying nests. It would therefore be helpful to know how far away a hornet’s nest could be from an afflicted apiary, a distance that will be affected by flight capacity.

To our knowledge, the flight capacities of *V. velutina* workers have never been studied. Here, we examined them in the lab because it is difficult to track hornets in the field, and indirect methods (e.g., classic mark-recapture methods) are not well suited to worker movings, or difficult to apply to them. Previous studies have used flight mills to characterize the flight capacities of insects and have discussed their relevance [18, 19]; while it is clear that they cannot fully capture natural flight dynamics, they nonetheless yield useful data. Flight mills create very artificial conditions: the insects do not experience the external stimuli that normally influence flight. They are incited to fly continuously because their legs have no support upon which to rest, and there are no flight-ending signals, such as the presence of food. Consequently, it is generally considered that flight mills measure maximum flight capacity. These caveats aside, flight mills provide abundant and precise data on flight activity that would be very difficult to obtain otherwise. They are also useful for studying the effect of different factors on flight under controlled conditions. However, caution must be taken when seeking to interpret these results in the context of natural flight. Most notably, because insects on a flight mill must overcome flight mill resistance but do not bear the load of their own mass, the results could be underestimated [20] as well as overestimated [21]. The ultimate effects of these potential biases are almost entirely uncharacterized. A few studies have compared tethered and free flight, but they only looked at initial flight [20, 22]. Other studies found that flight mill data are consistent with field data [19, 20, 23], and research comparing interspecific flight patterns in noctuid moths showed that results obtained using flight mills were consistent with current knowledge of dispersal abilities in nature [24]. In another study involving *Monochamus galloprovincialis* [25], flight capacities measured using flight mills were used to estimate the parameters of a model of a mark-recapture experiment, and the results of the model were consistent with the results of the experiment.

Here, we used flight mills to characterize the flight capacities of *V. velutina* workers. Our initial goal was to estimate colony foraging radius, but we also obtained a large amount of additional data about different aspects of *V. velutina* flight.

## Materials and methods

### Sampling and experimental design

The study lasted two years (2012–2013); it was carried out partially in the summer but mainly in the autumn, which is when hornet foraging activity is greatest [26, 27]. *Vespa velutina* workers were sampled in the Loire Valley (central France); they were either sampled from nests (Tours) or captured while foraging (near Orléans). In the former case, the insects were anesthetized with ethyl ether.

In 2012, we focused on analyzing daily flight capacity over time (i.e., worker post-sampling lifespan). In this experiment, half the hornets came from a nest in Ligueil (Department of Indre and Loire) sampled on September 4. The other half were hornets that had been foraging in La Ferté-Saint-Aubin (Department of Loiret) that were sampled September 10–19. The hornets were placed in individual terrariums, which were kept in the dark in climatic chambers maintained at 16 °C to limit wing damage related to insect agitation. They had access to unlimited water and honey. Their flight capacities were tested using flight mills (see below). Because we had a limited number of flight mills, we had to randomly select hornets for testing. However, to the greatest extent possible, those hornets were tested each day until they died (except weekends). When a new flight mill became available, an additional hornet was randomly selected for testing. A total of 88 workers were sampled, and 44 of them were tested.

In 2013, we focused on seasonal variation in worker flight capacity. Consequently, we sampled hornets at different time points and then tested them for an approximately two-week period. Sampling took place on July 18 (from a nest in Chinon, Indre and Loire) and on August 29–30, September 12–13, September 26, October 17, October 31, and November 8 (foragers from La Ferté-Saint-Aubin, Loiret). They were kept under laboratory conditions and tested as described above. A total of 225 workers were sampled, and 112 of them were tested.

### Flight mill design

**Fig 1:**
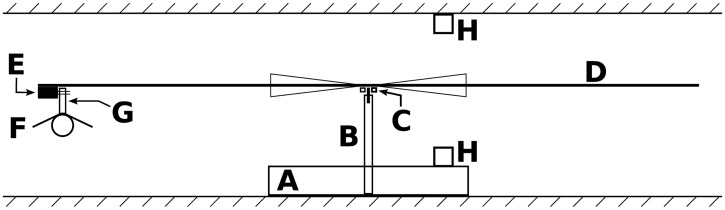
Flight mill design. A: support stand, B: shaft, C: ball bearing, D: arm with paper triangles, E: foam block, F: hornet, G: tether, H: infrared emitter and detector. Image created with Inskape 0.48.5 software (https://inkscape.org/).

We designed a flight mill to test the flight capacities of hornets, drawing on previously described models ([28]; Fig 1). The base of the flight mill was a cast iron support stand (Fisher Scientific 6693C) into which we screwed a threaded shaft (10 mm in diameter, 10 cm in height). A hole was drilled into the top of the shaft, and we inserted a 3-mm pin upon which a miniature ball bearing (RS Components SAS, NMB DDRF830ZZRA5P24LY121) was placed. A carbon-fiber arm (2 mm in diameter, 66 cm in length) was then attached to the ball bearing; the ball bearing minimized friction, allowing the arm to rotate easily. Triangular pieces of paper were taped to the center of the arm—they helped trigger the motion detection system. A small foam block was glued to one end of the arm using cyanoacrylate glue (Diall^®^). Two tiny pins were left in the foam and were used to secure the insect tether to the apparatus. This foam block was located such that the flight mill’s radius was 318.3 mm, meaning that an insect traveled 2 m per lap.

Arm movement was monitored using a computerized system. An infrared emitter and detector (RS Components SAS, Honeywell SPX1189-002 and SPX1189-003) were placed above and below the arm, respectively. They were connected to a PCI board (RS Components SAS, Arcom APCI-IB40) installed in a computer. When the arm cut the beam between the emitter and the detector (which the paper triangles helped ensure), an electrical signal was sent to the PCI board. An application written in C++ detected and time-stamped these signals, and wrote the resulting data in files (named logs below). Insect flight activity was thus precisely recorded.

The flight mills were kept in the laboratory. They were placed on shelves, and each was lit by an 8V fluorescent lamp (RS Components SAS, PHFP63A). Light intensity was low as compared to that in the field, but it was bright enough to allow hornet flight. Sixteen flight mills were used in total; they were located in two adjacent rooms.

The hornets were tethered to the flight mill using tethers. A tether consisted of a piece of cardboard (∼0.7 mm thick, 3 cm long, 4 mm wide), with a small piece of foam glued to one end using cyanoacrylate glue (Diall^®^). Before a flight test, the hornet was anesthetized using carbon dioxide, and the foam-free end of the tether was glued on the top of the hornet’s thorax using neoprene adhesive gel (Diall^®^); the foam end of the tether was placed above the insect’ head. Special care was taken to avoid putting glue on the wings. The foam on the tether was secured to the pins in the arm’s foam block, tethering the hornet to the flight mill.

### Flight tests

Hornets to be tested were equipped with tethers the day before or in the early morning of the test. The tether generally stayed glued to the insect for several days. It was replaced when it was lost.

The flight tests were performed during the day because *V. velutina* is generally described as being diurnal. Also, we saw that hornets stop flying when the flight mills were placed in the dark. Before each flight test, the hornet was weighed. It was then tethered to a flight mill and allowed to fly freely. No attempt was made to stimulate flight. After an hour, we gave the hornet a cotton pellet soaked in dilute honey (a 30% honey water solution); it was allowed to feed for 15 min before the pellet was removed (if it had not been dropped before then). This sequence of events was repeated for the duration of the flight test; the intent was to simulate feeding in the nest. However, we never interrupted a flight phase, so insects were not fed until they stopped flying. The flight tests ended in late afternoon, before a feeding event. We always tried to stop the flight tests during a rest phase, so some tests ended later because the hornets were still engaged in active flight. At the test’s end, the hornet was untethered, weighed, then returned to its terrarium. The flight tests were performed at room temperature (20 °C to 25 °C). A total of 509 flight tests were performed in 2012 and 466 were performed in 2013. They generally lasted about 7 h. However, some flight tests (23 in 2012, 14 in 2013) were designed to run from sunrise to sunset (full-day tests), so they lasted about 12 h.

### Data analyses

Scripts were developed to analyze the logs recorded by the flight mill computerized system. They divided the log of each flight test into different phases. Active phases (jump and flight phases, see below) occurred when hornets were moving and were separated by rest phases. The scripts also detected and excluded erroneous records, which sometimes appeared. We distinguished jump phases (i.e., very short flights) from “true” flight phases; the former have no functional significance from the perspective of *V. velutina* foraging activity and could easily have been artifacts. As no objectively defined limits emerged from the data (S1 File), we arbitrarily defined flight phases as the phases during which hornets covered at least 10 m. Similarly, we arbitrarily defined rest phases as the phases during which hornets seemed not move for at least 5s. For each phase, the scripts computed distance, duration and speed.

All the statistical analyses were performed using R software (version 3.3.3) [29]. It was also used to generate data figures. All tests were performed with a 95% confidence level. When the data were normally distributed, means and standard deviations are given. These data were analyzed using standard ANOVAs. When the data were not normally distributed, medians and quantiles/percentiles are given. These data were analyzed using Kruskal-Wallis rank sum tests. As each hornet was tested several times, statistical tests could be biased due to autocorrelation. To deal with this issue, flight test data were summarized per hornet before to be statistically tested. If not usable, we used a mixed model ANOVA including the insect identity as a random effect; if appropriate, flight test rank was also used as covariate; p-values were given for the tested variables. Differences in survival data were tested using Kaplan-Meier estimator and log-rank test. Uniformity of flight phases distribution throughout the day was tested using a Pearson Chi-squared test. We tried to fit variable distribution with known theoretical distributions, and the best ones are indicated in figures.

## Results

The experiments in 2012 and 2013 had different objectives and sampling designs, but some of data collected were similar. Here, we chose to present the data according to experimental objective, but we always verified that similar data from the other experiment (if available) gave consistent results.

### Daily flight capacity

**Fig 2:**
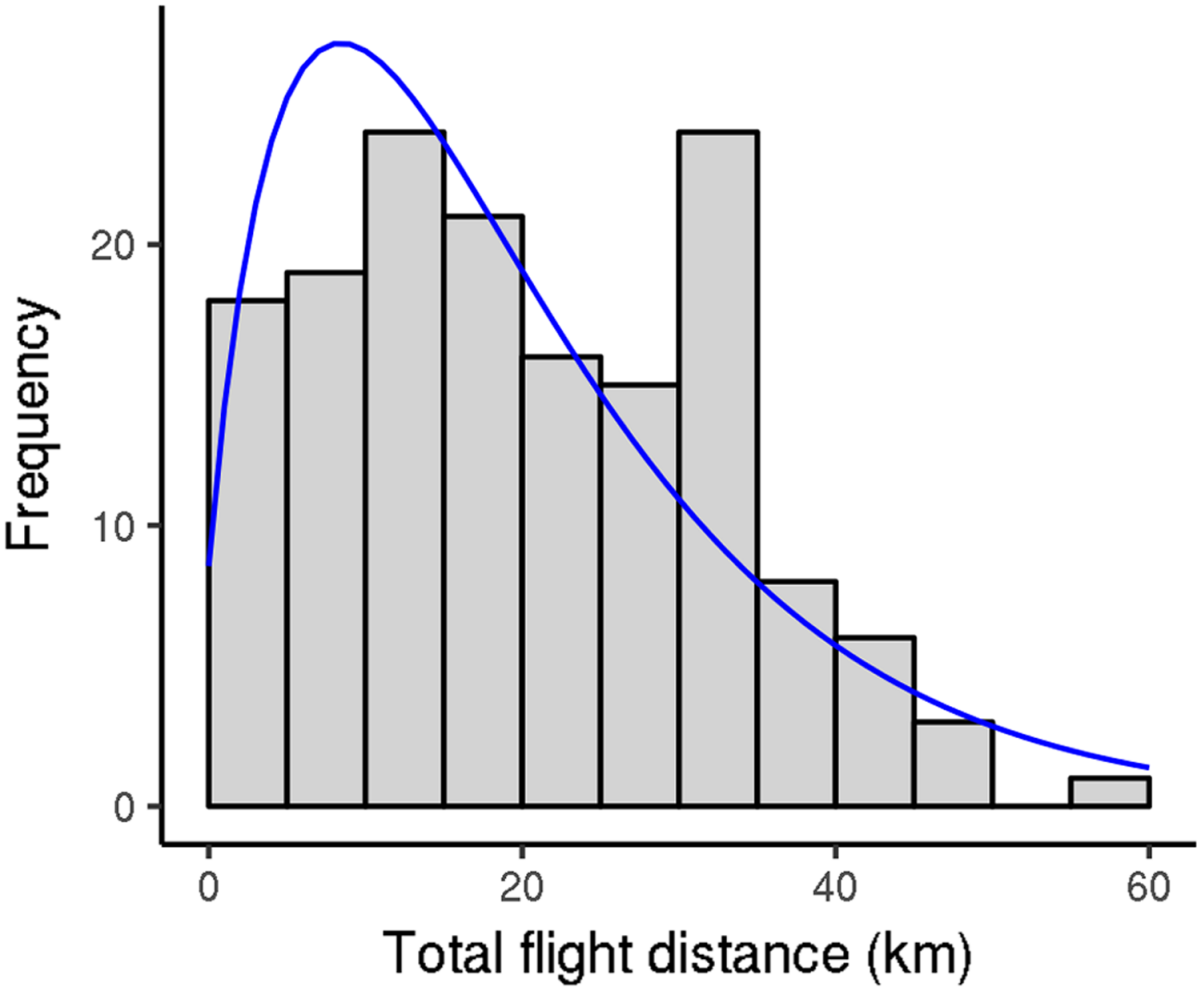
Distribution of the total distances covered by *V. velutina* workers during flight tests (2012 experiment). Fitting curve: negative binomial distribution, size 1.82, mean 19.9. Only the first five flight tests were considered for each worker.

**Fig 3:**
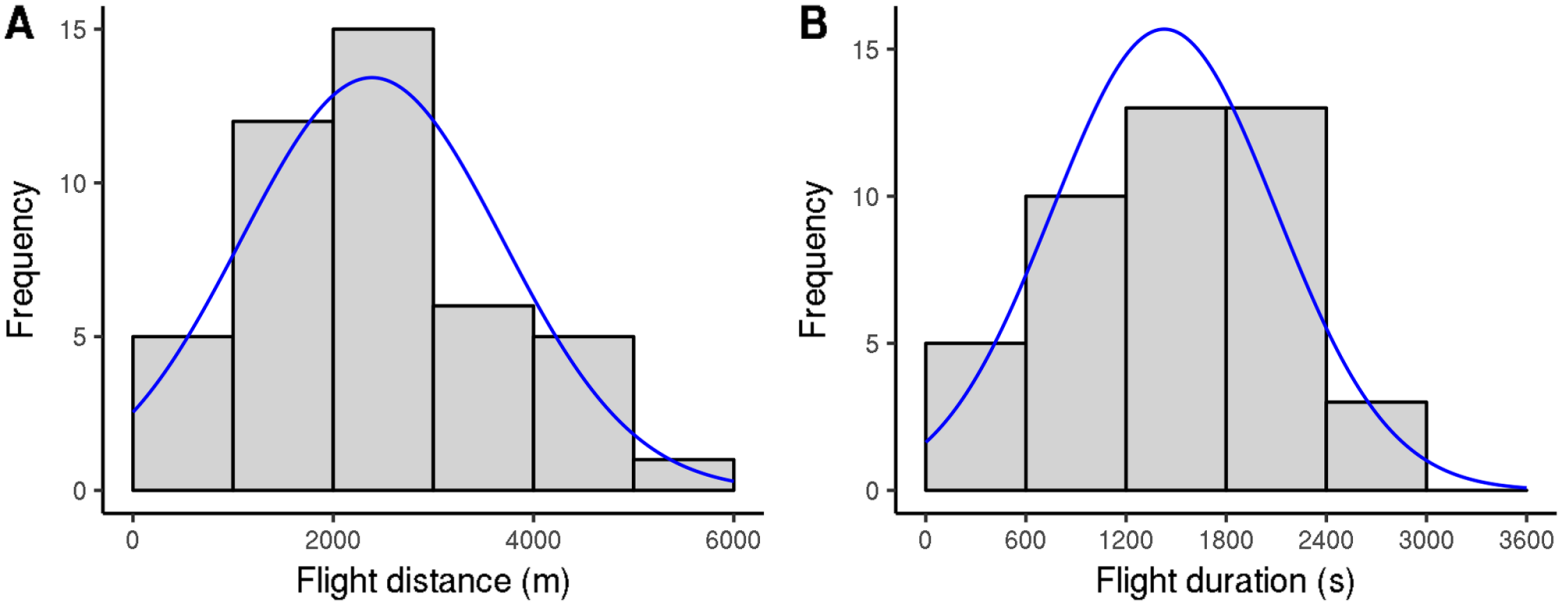
Flight capacities of *V. velutina* workers, expressed per hour of flight test (2012 experiment). A: Distribution of flight distance; fitting curve: normal distribution, mean 2386, standard deviation 1308. B: Distribution of flight duration; fitting curve: normal distribution, mean 1429, standard deviation 672. Only the first five flight tests were considered for each worker.

**Fig 4:**
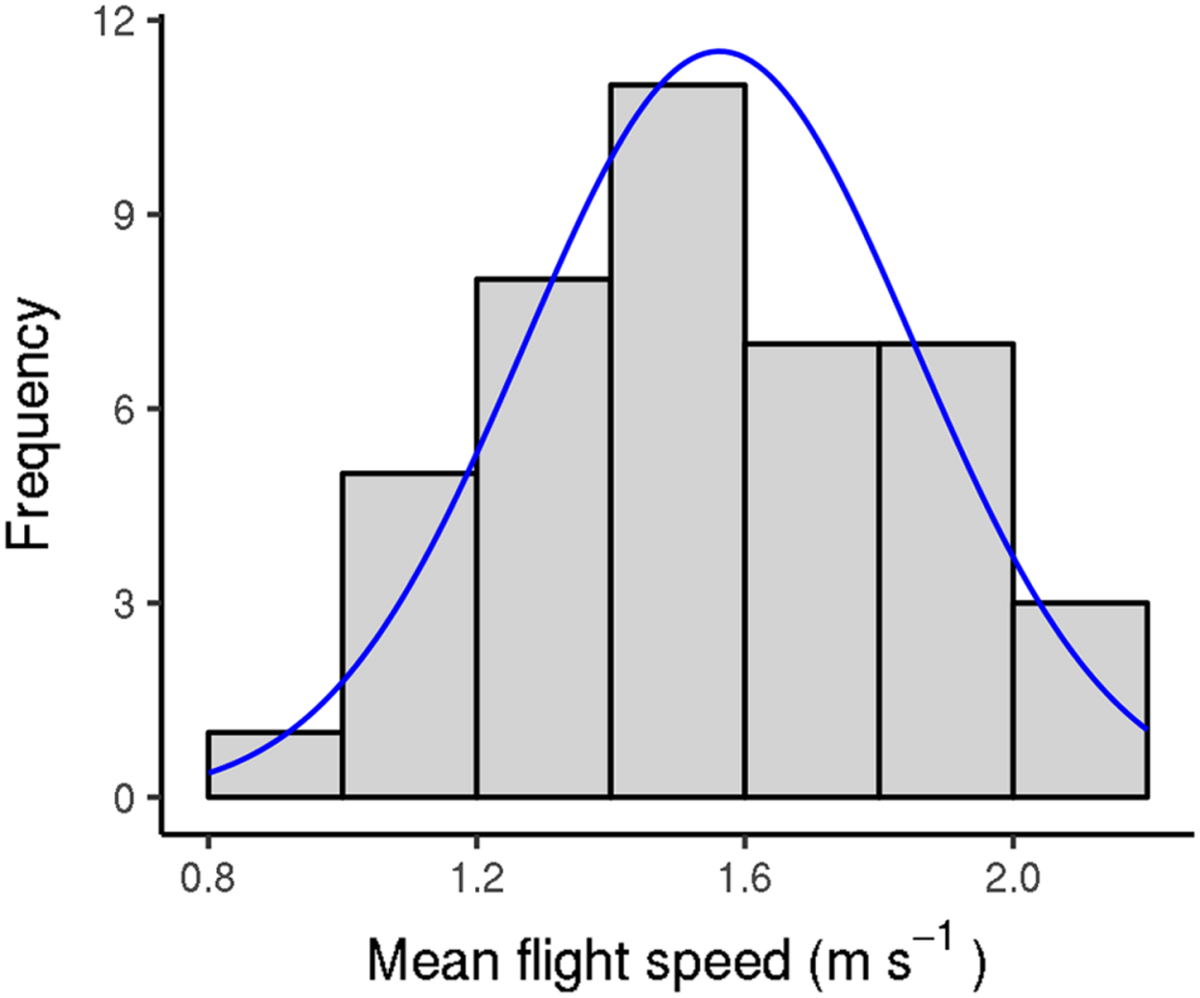
Distribution of *V. velutina* workers’ mean flight speed (determined using flight phases only) during flight tests (2012 experiment). Fitting curve: normal distribution, mean 1.56, standard deviation 0.291. Only the first five flight tests were considered for each worker.

Recently sampled *V. velutina* workers were usually able to fly 10km to 30km during each flight test, although there was a large amount of variation (Fig 2; 90% flew less than 37 km). The way in which we structured the flight test induced variation in their duration. To limit the effects of such disturbance, we time-standardized flight durations and distances by dividing their values by flight test duration (in hours). Similarly, because flight capacity changed as hornets aged (see below), we focused on the first five flight tests for each hornet, which generally took place in the week following its capture. In 2012, *V. velutina* workers flew (2386 ± 1323) m per hour on average; however, there was tremendous variation: values ranged from 0 m to 5285 m (Fig 3A). Flight duration was roughly proportional to flight distance, so mean flight duration was (1429 ± 679) s per hour (40% of a testing hour was spent flying); it ranged from 0 s to 2510 s (Fig 3B). Mean flight speed was (1.56 ± 0.29) ms^−1^; it displayed less variation than flight distance and duration (range 0.90 ms^−1^ to 2.10 ms^−1^; Fig 4). In the few full-day tests, the flight distance per hour was a little smaller than it was in the shorter-day tests: (1928 ± 1218) m versus (2168 ± 1218) m, p-value for flight test type = 0.047 (mixed model ANOVA including the insect identity and flight test rank as covariates; for this test, we used only workers that performed both full-day and shorter-day tests, and included data for the first 10 flight tests for each insect so as to include most full-day tests). Worker mass generally decreased with flight duration over the course of flight tests. Again, there was a large amount of variation (S2 File). Mass loss or gain could be considerable, representing up to 40% or 50% of the hornet’s initial mass (on average, hornets lost (15 ± 17)% of their initial mass). No difference was seen between workers sampled from nests and workers captured while foraging (p-values 0.37, 0.34, and 0.90 for distance flown per hour, flight duration per hour, and mean speed, respectively). All results were similar in 2013.

**Fig 5:**
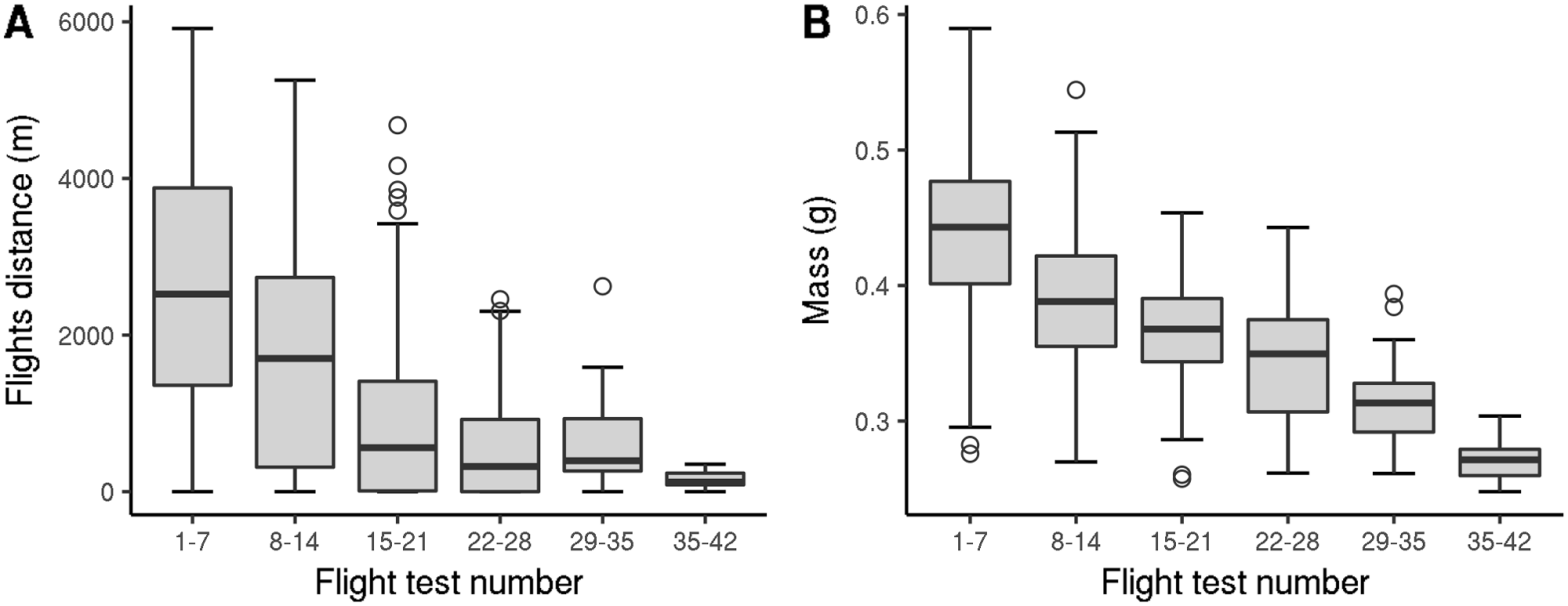
Changes in *V. velutina* worker flight-related characteristics over the course of successive flight tests (2012 experiment). A: Flight distance per hour. B: Mass at the beginning of the flight test. ANOVA p-value for flight test number = 3.3 × 10^−7^ and 1.8 × 10^−5^ for A and B, respectively; workers that performed less than 14 flight tests were excluded to avoid singularities in ANOVA. As no workers captured while foraging were tested more than 27 times, the data from flight test number 29 was obtained from workers sampled from nests.

Some hornets died when they were captured, and many other hornets died when they were anesthetized and extracted from their nests. These insects were excluded from the survival data. Workers could survive several weeks under laboratory conditions, with some living more than two months (Table 1). Median post-capture longevity was greater for workers sampled from nests than for workers captured while foraging (Table 1; p-value = 0.000582). Worker flight distances were stable for a few days at a time but decreased over a worker’s remaining lifespan (Fig 5A; flight duration showed a similar pattern). Mean flight capacity was lower for workers captured while foraging than it was for workers sampled from nests, but these differences seemed to be never significant (mixed model ANOVA was not usable due to singularities; means in each lifetime period did not significantly differ). Even after their flight capacities had weakened, workers sampled from nests could survive for several more days (Fig 5A; up to 25 days) while workers captured during foraging died quickly (none were tested more than 27 times). Worker mass decreased over time (Fig 5B). Means were similar for workers captured during foraging and workers sampled from nests, but while the latter simply attained a lower mass, the former died (Fig 5B). Flight speed did not significantly change over time (ANOVA p-value for flight test number = 0.36).

**Table 1:** Post-capture survival and number of flight tests for *V. velutina* workers according to sample type (taken from nests or captured while foraging, 2012 experiment).

Origin	Frequency	Survival (days)		Number of flight tests	
		Median	Max	Median	Max
foraging	30	26	47	4	27
nest	22	41.5	87	13	41

Insects killed upon capture were excluded. Frequency: number of workers.

**Fig 6:**
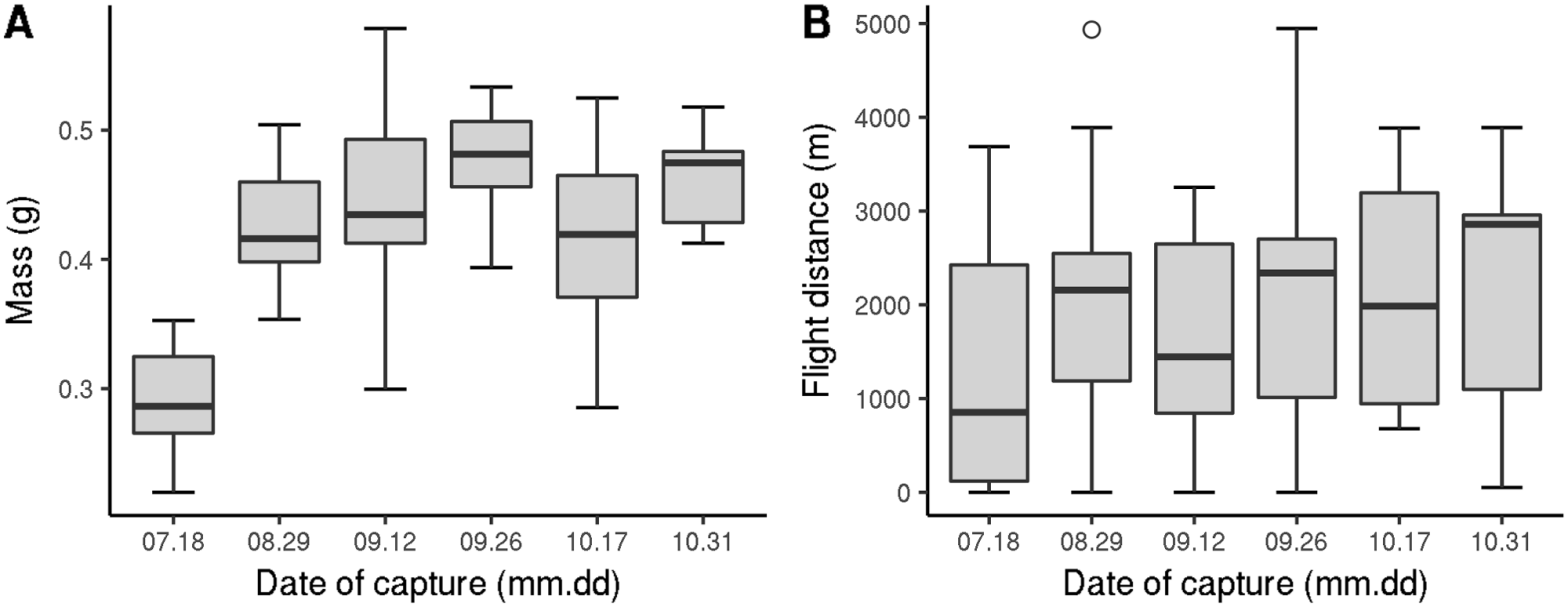
Seasonal changes in *V. velutina* worker flight-related characteristics (2013 experiment). A: Mass at the beginning of the flight test. B: Distance per hour.

The flight-related characteristics of *V. velutina* workers varied by season. Their mass was lower in early summer (July) than in late summer and autumn (August–October); it appeared stable during the latter months (Fig 6; Table 2). Their flight capacities (i.e., flight distance, duration, and speed) reflected a similar pattern. The lower mean flight capacity in early summer seemed to be driven by the fact that a higher proportion of insects flew markedly less, which can be seen in the difference in the first quartiles, which is much greater than the differences in the medians and maxima.

**Table 2:** Variation in flight-test-related characteristics of *V. velutina* workers according to capture period (2013 experiment).

Capture period	Mean initial mass (g)	Flight distance per hour (m)			Flight duration per hour (s)			Mean speed (ms^−1^)
		Q1	Median	Max	Q1	Median	Max	
July	0.316 ± 0.061	19	294	3687	35	436	2331	0.95 ± 0.38
Aug–Oct	0.447 ± 0.057	957	2077	4947	688	1466	2810	1.42 ± 0.31
	2.2 × 10^−16^		0.00016			0.00091		1.2 × 10^−7^

Q1: first quartile. Last row contains p-values for mean or median comparison.

### Flight phase distribution

**Fig 7:**
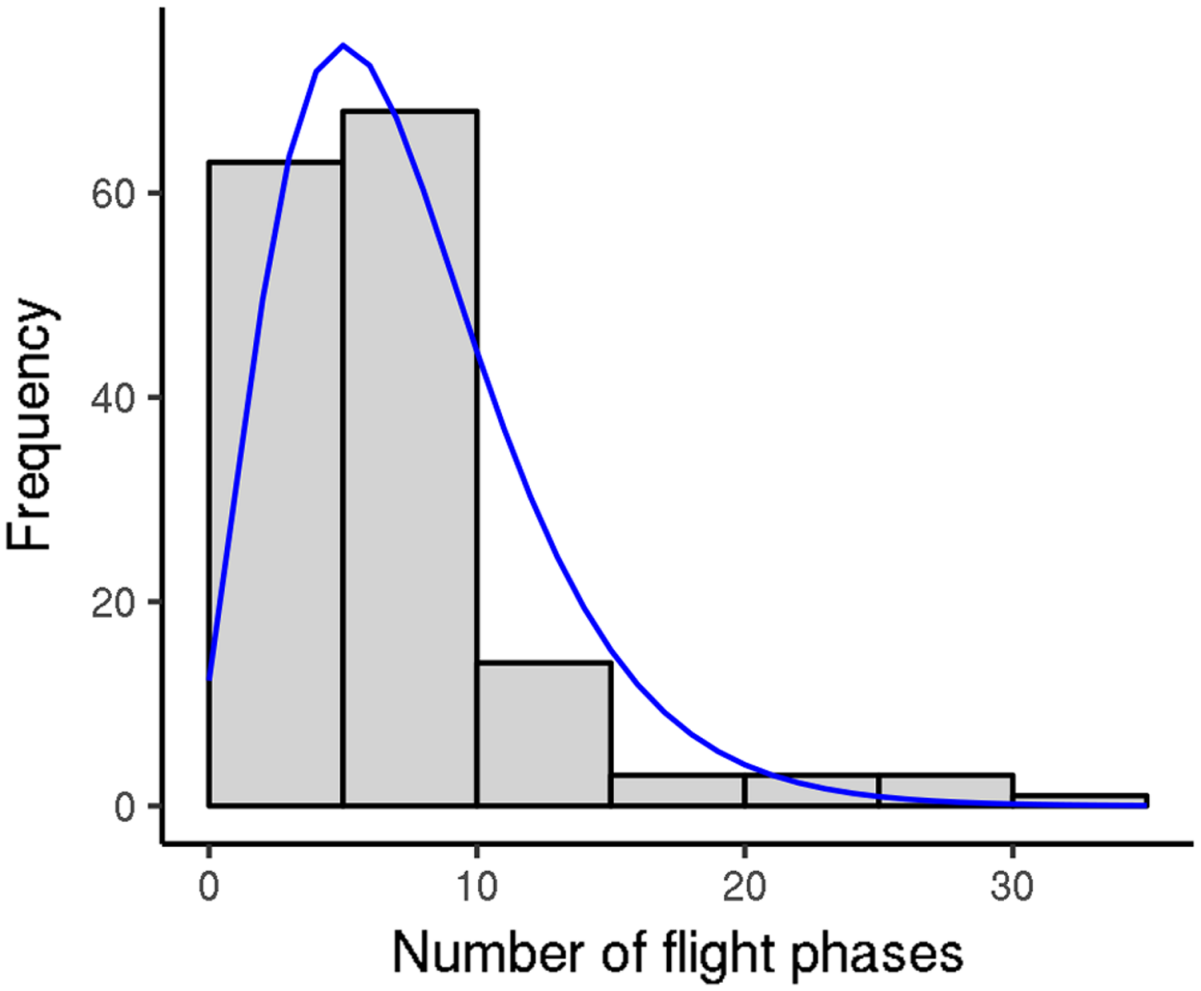
Number of flight phases performed per flight test by *V. velutina* workers (2012 experiment). Fitting curve: negative binomial distribution, size 3.83, mean 7.48. Only the first five flight tests were considered for each worker.

**Fig 8:**
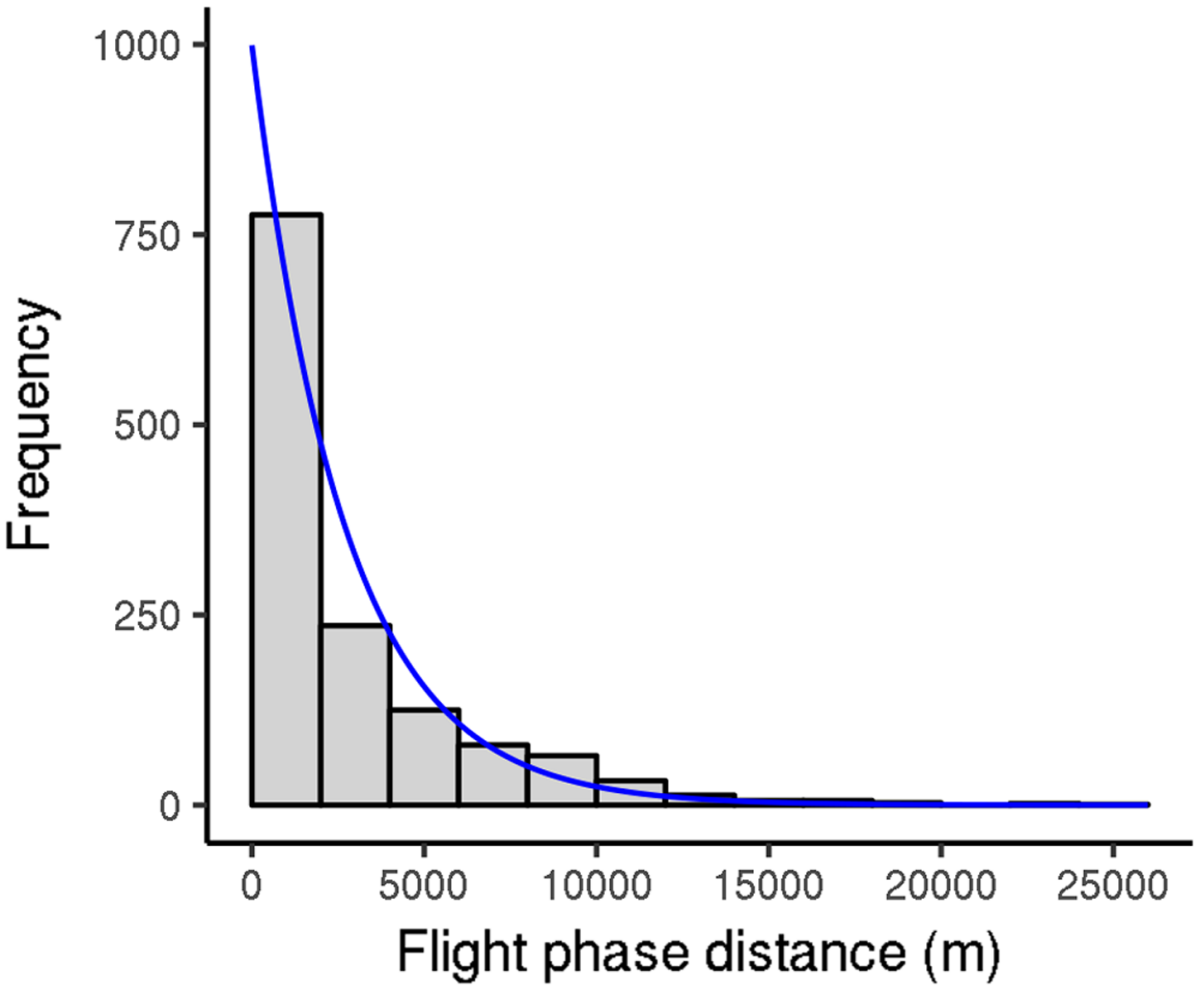
Distribution of flight phase distance for *V. velutina* workers (2012 experiment). Fitting curve: exponential distribution, rate 0.372 (computed in kilometers). Only the first five flight tests were considered for each worker.

**Fig 9:**
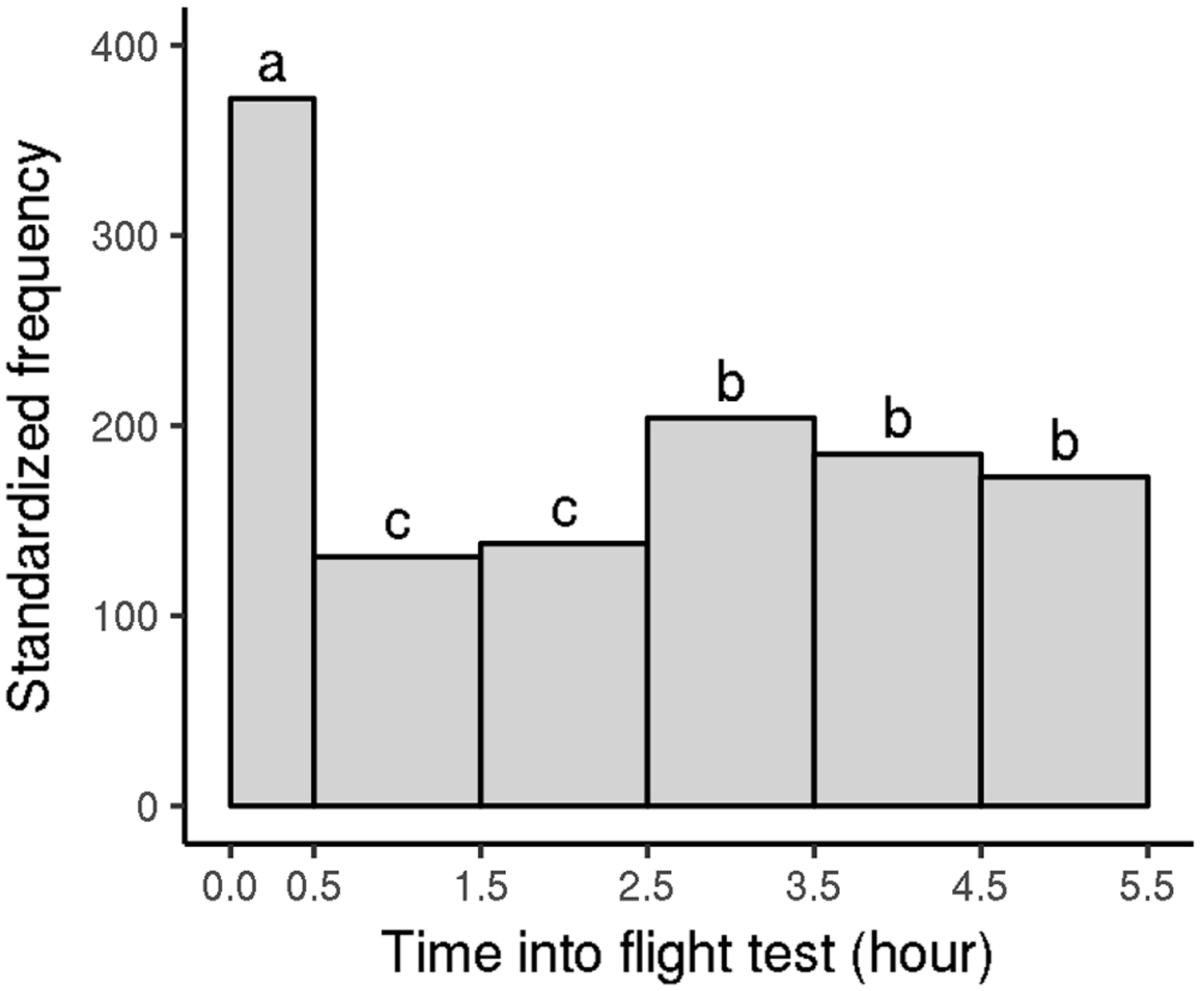
Distribution of flight phases performed by *V. velutina* workers according to the time into the flight test (2012 experiment). Only the first five flight tests were considered for each worker. Flight phases were distributed according to their time of beginning. Because some flight tests finished after around 5.5 h, when others ran longer, any flight phases beginning after 5.5 h were excluded to avoid biasing the overall results. Frequencies were standardized to reflect the number of phases per hour of flight test. Differences in letters indicate significant differences in frequencies; global *χ*^2^: p-value < 2.10^−16^; *χ*^2^ between late morning (0.5–2.5 h) and afternoon (2.5–5.5 h): p-value = 0.0015).

We also analyzed the distribution of flight phases during flight tests of *V. velutina* workers from the 2012 experiment; as above, we focused on the first five flight tests for each hornet. During each flight test, workers generally performed several flight phases, although there was marked variation (Fig 7; mean = 7.5 flight phases, median = 6 flight phases). They also performed several jump phases (mean = 20), but the percentage contribution of jump phases to overall flight activity was minimal (about 0.2%). The distance and duration of the flight phases were highly variable (Fig 8; Table 3: early category). Many flight phases were rather short, but around 25% of them covered at least 4000 m, 10% of them covered at least 7900 m, and the maximum distance covered was 26 000 m, which corresponded to 4 h of continuous flight. When *V. velutina* workers carried out flight phases of above-median distances, they performed 4.2 ± 1.8 such phases per flight test (max = 11). Similarly, those that carried out flight phases of above-third-quartile distances performed 2.8 ± 1.4 such flight phases per flight test (max = 7). Finally, when workers carried out flight phases with distances above the 90th percentile, they generally performed just one such phase per flight test (median = 1, max = 4). The flight and jump phases were separated by rest phases of short but highly variable duration (mean = (741 ± 1318) s, median = 22 s, third quartile = 1065s). Many flight phases occurred very early in the flight test; their number then dropped off before increasing again (Fig 9). Flight phase distance did not vary much throughout the day, except that flight phases during the first 30 min of the flight test were longer (medians = 4130 m vs 2192 m, respectively; p-value = 0.00013). These results were similar when the 2013 data were examined.

**Table 3:** Descriptive statistics for the distance and duration of *V. velutina* worker flight phases (2012 experiment).

Tests	Feature	Mean	Median	Q3	P90	Max
Early	Distance (m)	2724 ± 3526	1351	4018	7932	25900
	Duration (s)	1620 ± 1955	914	2520	4397	14280
Late	Distance (m)	1493 ± 2358	457	1902	4480	25070
	Duration (s)	923 ± 1333	376	1206	2625	11570

Early: only the first five flight tests for each worker were considered; Late: the first five flight tests for each worker were excluded; Q3: third quartile, P90: 90th percentile. Comparison of medians between early and late tests: both p- value < 2.10^−16^.

As *V. velutina* workers aged, the distribution of flight phases remained fairly unchanged. While the number of flight phases per flight test did not vary, flight phase distance and duration decreased (Table 3). In the 2012 experiment, when the first five flight tests were excluded, we observed that when *V. velutina* workers carried out flight phases of above-median distances, they performed 4.8 ± 26 such flight phases per flight test (max = 16). Similarly, those that carried out flight phases of above-third-quartile distances performed 3.1 ± 1.7 such phases per flight test (max = 8). Finally, when workers carried out flight phases with distances above the 90th percentile, they generally performed two such phases per flight test (median = 2, max = 6).

Some parameters of flight phases distribution differed between early summer (July) and late summer/autumn (August–October). In early summer, the number of flight phases per flight test was greater and there was a larger proportion of flight tests with many flight phases (median = 24 vs 16, respectively; Q3 = 95 vs 24, respectively; p-value = 0.0054). In addition, flight phase distance was shorter and there was a larger proportion of very short flight phases (median = 604 m vs 1518 m, respectively; Q1 = 57 m vs 1034 m, respectively; p-value = 0.00077).

## Discussion

This study demonstrates that flight mills can reveal interesting information about insect flight. Even though flight mill studies occur under laboratory conditions, they allow the collection of abundant data on the general flight capacities of *V. velutina* workers.

Overall, we observed a considerable amount of variation in flight capacity. This result has been seen before in other insect groups, including beetles [30], moths [19, 31] and honeybees [32]. However, it was nonetheless surprising at first glance because the *V. velutina* population in Europe is very genetically homogeneous due to founder effects [8], and it is certain that some of the workers we used were even more related (e.g., hornets sampled from a given nest were at least half-sisters). That said, other sources of variability exist, such as worker age and behavior. We did not control for worker age, which clearly reduced flight capacity and thus contributed to flight variability. It is also possible that hornet workers reversibly behaviorally specialize in short or long flights, like honeybee workers [32]. Regardless of the origin of this variability, our analyses must account for it, especially because it has an impact on statistical power.

In this study, *V. velutina* worker flight was essentially a succession of flight phases interspersed with rest phases during which wasps might rest and feed. This pattern is consistent with the biology of the hornets in nature: in the field, workers (except the youngest ones) mainly forage. Each day, they make several foraging trips, returning to the nest in between to rest and obtain food from larvae [33]. We did not observe major changes in flight phase distance over the course of the day, with the exception that longer flight phases took place very early on. This pattern could be the result of hornets being in better condition after resting overnight. However, it could also be an artifact, a response by hornets to tethering. It could also be a combination of both. Many hornets began the flight test with a flight phase. This first phase was probably a response to tethering. We also observed that the hornet initiated a greater number of flight phases during the second half of the flight test (i.e., in the afternoon). This pattern is consistent with field observations, including ours (Mercier and Darrouzet), which indicate that hornet activity is generally higher around or after midday [34]. This trend is probably due to changes in external conditions, mainly temperature. We did not observe a decrease in flight activity near the end of the flight test, but temperature changes slowly in the laboratory and the flight tests generally ended before it could do so.

*Vespa velutina* workers appear to have good flight capacities. They were able to fly continuously for about 40% of the day. By multiplying hourly flight capacity by day length, we can estimate daily flight capacity. In September, days last 12 hours and the distance traveled is estimated to be about 25 km per day (taking into account the tiredness revealed by full-day flight tests). However, this estimate may or may not reflect performance under field conditions, where flight activity is influenced by temperature and other environmental factors. The flight capacity we observed here for the yellow-legged hornet is much higher than those observed under similar conditions for beetles that are relatively poor flyers (*Monochamus galloprovincialis*: 2 km per day [18]; *Rhynchophorus ferrugineus*: 2.6 km per day [30]). It is, however, similar to that of honeybees (several tens of kilometers per day [35]). This estimate is also consistent with the biology of *V. velutina* workers, which spend a large proportion of their lives flying.

The mean flight speed for *V. velutina* workers was 1.56 ms^−1^, which is similar to those of the beetles mentioned above [18, 30]. However, it is much lower than the speed traveled in free flight by honeybees (3 ms^−1^ to 9 ms^−1^ [35, 36]) and European hornets (up to 6 ms^−1^ [37, 38]). Our past observations in the field have been more consistent with the the latter results. Even if the flight speed of foraging *V. velutina* workers is poorly characterized in the field, it seems to be underestimated on flight mill. Our study is not the first, though, to see such phenomenon (e.g., [20] or [37]). It is possible that the hornets were slower on the flight mill because of friction within the apparatus, although lighter insects were tested using the same flight mill without problem. A more probable explanation is that the hornets slowed down because of disruptions in optical flow (the visual motion of environmental features experienced by the eyes as an individual moves) as the hornets flew. This issue has been observed in honeybees [39]. The underestimation of flight speed might have affected flight capacity. That said, flight capacity depends largely on energy. While the relationship between flight speed and energy consumption is not well characterized in insects, it has generally been observed that the rate of energy consumption increases with speed [40–42]. Consequently, energy consumption may depend more on the distance traveled than on speed, meaning that flight speed can only minimally bias flight capacity.

Some *V. velutina* workers lost a lot of mass during the flight test, as much as half of their starting mass. As could be expected, loss of mass, which was basically null for non-flying hornets, increased with flight activity. It was the result of energy consumption and water loss during flight, as well as of reduced access to food, since the hornets were not fed as they flew. Hornet mass did not differ between consecutive pairs of days, indicating that hornets were able to recuperate at night. From this perspective, the rearing conditions seemed well suited to hornets. However, as the hornets rarely flew for more than an hour, an increase in feeding frequency could limit the loss of mass by reducing the span of time between a flight phase’s end and the opportunity to feed; this might improve workers’ flight capacities.

*Vespa velutina* workers survived several weeks under our rearing conditions. Their age at capture was unknown, so this survival duration was an obvious underestimation of lifespan. The underlying reason for the difference in survival between workers sampled from nests and workers sampled while foraging is unclear. It might be due to the presence in the nest of the youngest workers, who do not go out. However, the pre-foraging period is very short in [g]Vv species, generally two to four days [33, 43], even if we do not have a species-specific estimate for *V. velutina*.

It should also be noted that the longevity we observed likely represents potential lifespan in controlled circumstances. Foraging is a dangerous activity because of the presence of predators and other mortal hazards. Consequently, most workers probably have a much shorter lifespan in nature than in the lab. Although actual mean longevity for the yellow-legged hornet is unknown, *Vespa* workers are generally thought to live just two or three weeks [33]. In our study, hornets suffered a significant physical decline after about two weeks of flight tests. These issues may have resulted from the normal aging process, or in whole or in part from a failure in our rearing procedure. If the decrease in flight capacity is related to aging, it could lead to the rapid death of hornets in the field. However, because their natural lifespan appears to be short, this phenomenon probably has little to no impact under field conditions.

The flight capacities of *V. velutina* workers did not display seasonal differences, except in early summer. This pattern could not be explained by sample type because it was not seen in workers obtained from the other nest. In early summer, many of a given colony’s workers will have hatched just after founding. At that point in time, food supply is likely limited by the colony’s small number of foragers. The hornets we collected in July were probably relatively malnourished, which would explain their small size [27, 33]. Our study shows that smaller worker size in early summer is associated with diminished flight capacity.

Estimating the foraging radius of *V. velutina* colonies would be helpful in control efforts because it would make it easier to find and destroy nests, which is currently the best method for dealing with this invasive pest. Foraging behavior may vary in complexity (e.g., dynamics may be different for departures and returns), but little is known about the topic in the yellow-legged hornet and such data cannot be obtained with flight mills. Here, we therefore estimated colony foraging radius using the simplest model possible: hornets were assumed to fly directly from their nest to a resource and immediately return along the same path, without significant resting or feeding. We also assumed that flight capacity is the only factor limiting the foraging radius. This model is probably quite appropriate for describing a hornet colony that is exploiting a well-established resource, such as an apiary. In this model, the foraging radius depends explicitly on the distance a worker can cover during a flight phase. As this distance varies among individuals, the foraging radius is statistically defined, and several radius estimates can be produced depending on the proportion of workers that can fly a given distance. According to our results, 50% of workers can cover 1400 m. Consequently, the median foraging radius is 700 m (because 1400 m = round trip). For 25% of workers, the estimated foraging radius is 2000 m, and some hornets are able to forage much farther than that. While these estimates rely on several untested assumptions and on data obtained under laboratory conditions, they are consistent with beekeepers’ observations and data on *Vespa mandarinia*, a larger hornet (usual foraging distance = 1000 m to 2000 m; max = 8000 m [43]). They are also rather similar to the foraging radii of various bees [44–46], including honeybees [47, 48]. Finally, our estimates are also consistent with previous field observations [49]—in that study, workers from an isolated *V. velutina* nest were monitored as they foraged, and most were seen to move 500 m to 700 m away from the nest. Some traveled up to 1150 m. However, the estimates obtained here need to be compared with more direct field measurements. The values we arrived at are theoretical; in the field, many factors could influence colony foraging radius. For example, temperature, photoperiod, and wind can all act on flight and thus on foraging distance. However, resource availability around the nest and existing obstacles also play important roles. Additionally, colony foraging radius could depend on resource type: more valuable resources could be worth a greater effort. Our data suggest that the foraging radius may be smaller in early summer (300 m and 700 m for 50% and 25% of workers, respectively). If so, young colonies may be in a precarious situation if local resources are scarce and locating nests could be easier during this period.

Here, we characterized the flight capacities of workers from an invasive population of *V. velutina*, which is very genetically homogeneous [8]. Indeed, this homogeneity has resulted in inbreeding depression [50]. Consequently, these flight capacities may well not be representative of those of other populations of this widely distributed species. However, the large amount of variability that we observed suggests that this species, or at least the invasive population in Europe, could partly adapt its flight capacities to meet colony needs. The hornet’s excellent flight capacity and its potential adaptability may have been important factors in the species’ success. These traits would allow a hornet colony to access resources present across a vast area and thus adapt to new conditions or new locations. Such capacities could also partly explain the great invasive potential of Vespidae: at least 2 *Vespa* species from among the 22 members of the genus are well-established invaders (*V. crabro* in North America and *V. velutina* in Europe and Korea), and *Vespula germanica* is invasive in several places all around the world [51].

Flight mills can yield abundant information on *V. velutina* flight capacity and, in particular, allow colony foraging radius to be estimated. However, it also gathers these data under artificial conditions, so it is important to confirm results using field experiments. The flight patterns we saw in the laboratory are consistent with what we know about hornet biology, but field data on flight capacities are lacking. We also need to gather more information about the environmental factors, such as temperature, that may shape *V. velutina* flight capacity.

## Supporting information

S1 File Determination of flight thresholds. (PDF)

S2 File *Vespa velutina* workers weight loss according to cumulative flights duration. (PDF)

S3 File Description of data files. (PDF)

S4 File Insects data. (CSV)

S5 File Flight tests data. (CSV)

S6 File Flight test phases data. (CSV)
