# Supplementary material for: Flight capacities of yellow-legged hornet (Vespa velutina nigrithorax, Hymenoptera: Vespidae) workers from an invasive population in Europe
Source: PLoS One. 2018 Jun 8;13(6):e0198597. doi: 10.1371/journal.pone.0198597 (PMC5993251; doi:10.1371/journal.pone.0198597)
Supplement: S1 File — (PDF) [file pone.0198597.s001.pdf]

Flight capacities of yellow-legged hornet (*Vespa velutina nigrithorax*, Hymenoptera: Vespidae) workers from an invasive population in Europe

Daniel Sauvard, Vanessa Imbault, Éric Darrouzet

Determination of flight thresholds

The scripts that analyzed the flight mill logs and divided each log into several phases needed to determine how to distinguish between flights, jumps (very short flight phases), and rests. The frequency of active phases showed a negative curvilinear relationship with phase distance, so there was no objective way of distinguishing between jumps and flights (Fig. 1; the distributions were similar regardless of the flight tests used or the distance range). Similarly, there was a negative curvilinear relationship between the frequency of delays between laps and delay length, so there was no objective way of distinguishing between slow jumps/flights and resting periods. We thus had to arbitrarily define limits.

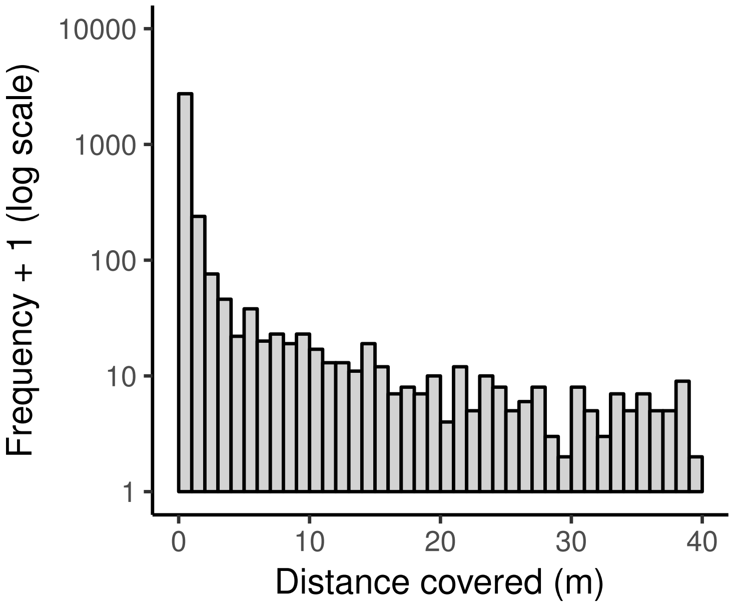

Fig 1. Frequency of short active phases ($\leq 40\,\mathrm{m}$) according to the distance covered (2012 experiment). Only the first five flight tests were considered for each worker.
