# Supplementary material for: Flight capacities of yellow-legged hornet (Vespa velutina nigrithorax, Hymenoptera: Vespidae) workers from an invasive population in Europe
Source: PLoS One. 2018 Jun 8;13(6):e0198597. doi: 10.1371/journal.pone.0198597 (PMC5993251; doi:10.1371/journal.pone.0198597)
Supplement: S2 File — (PDF) [file pone.0198597.s002.pdf]

Flight capacities of yellow-legged hornet (*Vespa velutina nigrithorax*, Hymenoptera: Vespidae) workers from an invasive population in Europe

Daniel Sauvard, Vanessa Imbault, Éric Darrouzet

Vespa velutina workers mass loss according to flight duration

During flight tests, *V. velutina* workers' mass generally decreased according to total duration of flight phases (Fig. 1). Results were similar according to total distance of flight phases, or with relative mass loss.

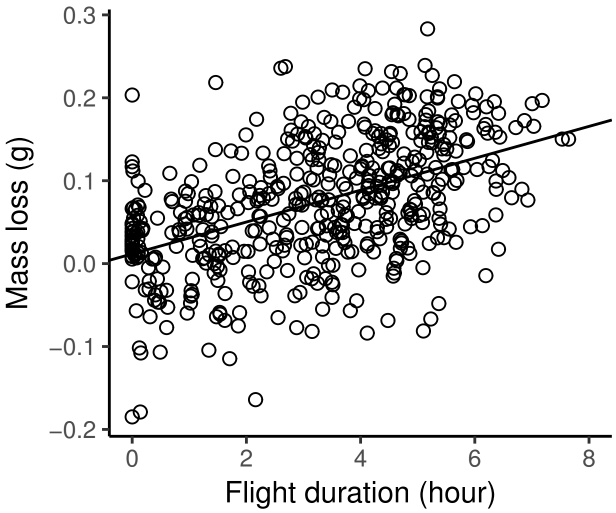

Fig 1. Mass loss of *V. velutina* workers during flight tests according to total duration of flight phases (tests from 2012 and 2013 experiments were pooled). Only the first five flight tests were considered for each worker. Regression line $y = 0.01167 + 0.01923 \times x$, $R^2 = 0.25$, p-value $< 2.10^{-16}$.
