# Supplementary material for: Flight capacities of yellow-legged hornet (Vespa velutina nigrithorax, Hymenoptera: Vespidae) workers from an invasive population in Europe
Source: PLoS One. 2018 Jun 8;13(6):e0198597. doi: 10.1371/journal.pone.0198597 (PMC5993251; doi:10.1371/journal.pone.0198597)
Supplement: S3 File — (PDF) [file pone.0198597.s003.pdf]

# Flight capacities of yellow-legged hornet (*Vespa velutina nigrithorax*, Hymenoptera: Vespidae) workers from an invasive population in Europe

Daniel Sauvard, Vanessa Imbault, Éric Darrouzet

## Data files

### S4_File.csv: data on insects used during the experiments

Fields:

- experiment, insect_type, insect_no: identication of the insect;
- arrival_date, arrival_mode: date of insect sampling, and field indicating whether it was captured during foraging ("capture") or sampled from a nest ("extraction");
- disposal_date, is_dead_at_disposal: date of insect disposal, and boolean indicating whether it died or was discarded;
- insect_nb_tests: total number of flight tests performed by the insect.

### S5_File.csv: data of daily flight tests

Fields:

- experiment, insect_type, insect_no: identication of the tested insect;
- arrival_week: seasonal group of the tested insect (mm.dd; only useful for 2013 experiment);
- insect_test_no: number of the flight test into the tested insect lifespan;
- is_long_test: boolean indicating whether flight test was full-day or shorter-day flight test;
- test_begin_date, test_begin_time, test_end_date, test_end_time: date and time of the beginning and the end of the flight test;
- test_duration: duration of the flight test;
- nb_flights: number of flight phases that occurred during the flight test;
- flights_duration, flight_mean_duration: total and mean duration of flight phases that occurred during the flight test;
- flights_length, flight_mean_length: total and mean distance of flight phases that occurred during the flight test;
- flights_mean_speed: mean speed during flight phases that occurred during the flight test;

- flights_duration_per_hour, flights_length_per_hour: total duration and distance of flight phases that occurred during the flight test, expressed per hour of test;
- nb_jumps: number of jump phases that occurred during the flight test;
- jumps_duration, jump_mean_duration: total and mean duration of jump phases that occurred during the flight test;
- jumps_length, jump_mean_length: total and mean distance of jump phases that occurred during the flight test;
- jumps_mean_speed: mean speed during jump phases that occurred during the flight test;
- nb_rests: number of rest phases that occurred during the flight test;
- rests_duration, rest_mean_duration: total and mean duration of rest phases that occurred during the flight test;
- insect_before_weight, insect_after_weight, weight_loss: mass of the tested insect before and after the flight test, and subsequent mass loss;

## S6_File.csv: data of phases of daily flight tests

Fields:

- experiment, insect_type, insect_no: identication of the tested insect;
- test_begin_date, test_begin_time: date and time of the beginning of the flight test;
- phase_no: number of the phase into the course of the flight test;
- phase_type: type of phase, ”flight”, ”jump”, or ”rest” (other values refer to utilitary phases);
- phase_begin_offset: phase beginning time from flight test beginning;
- phase_duration, phase_length: phase duration and distance;
- phase_mean_speed: hornet mean speed during the phase;
